# The tiny effects of respiratory masks on physiological, subjective, and behavioral measures under mental load in a randomized controlled trial

**DOI:** 10.1038/s41598-021-99100-7

**Published:** 2021-10-01

**Authors:** Robert P. Spang, Kerstin Pieper

**Affiliations:** Quality and Usability Lab, Institute of Software Engineering and Theoretical Computer Science, Electrical Engineering and Computer Science, Technical University of Berlin, Berlin, Germany

**Keywords:** Physiology, Psychology, Public health

## Abstract

Since the outbreak of the coronavirus disease (COVID-19), face coverings are recommended to diminish person-to-person transmission of the SARS-CoV-2 virus. Some public debates concern claims regarding risks caused by wearing face masks, like, e.g., decreased blood oxygen levels and impaired cognitive capabilities. The present, pre-registered study aims to contribute clarity by delivering a direct comparison of wearing an N95 respirator and wearing no face covering. We focused on a demanding situation to show that cognitive efficacy and individual states are equivalent in both conditions. We conducted a randomized-controlled crossover trial with 44 participants. Participants performed the task while wearing an N95 FFR versus wearing none. We measured physiological (blood oxygen saturation and heart rate variability), behavioral (parameters of performance in the task), and subjective (perceived mental load) data to substantiate our assumption as broadly as possible. We analyzed data regarding both statistical equivalence and differences. All of the investigated dimensions showed statistical equivalence given our pre-registered equivalence boundaries. None of the dimensions showed a significant difference between wearing an FFR and not wearing an FFR.

Trial Registration: Preregistered with the Open Science Framework: https://osf.io/c2xp5 (15/11/2020). Retrospectively registered with German Clinical Trials Register: DRKS00024806 (18/03/2021).

## Introduction

Throughout the COVID-19 pandemic, most countries quickly adopted – amongst others – face coverings as a measure to protect the general public. Face coverings can be roughly categorized into face masks (including cloth face coverings), surgical masks, and respirators. According to the FDA, face masks are coverings for the nose and mouth and do not meet filtration efficiency levels (not intended for medical purposes). In contrast, surgical masks meet several protection standards and are considered a medical device. However, their loose fit does not provide complete protection from contaminants^1^. The tight-fitting filtering facepiece respirators (FFRs) such as N95 (US) provide specific filtration efficiencies (at least 95% of small (0.3-micron) particles) and thereby higher virus protection^1,2^. Additionally, surgical masks and FFRs are disposable and should therefore be replaced regularly^1^.

Surgical masks and FFRs diminish person-to-person transmission of the SARS-CoV-2 virus^3^. Aerosols better diffuse around one's head by redirecting the exhaled emissions^4^. This process reduces exposures (if other measures such as a sufficient distance are adopted as well)^5,6^. The scientific background at present shows that N95 FFRs without a valve also filter particles, droplets, and aerosols in the in- and exhaled air, which reduces the risk of infection for the person wearing such an FFR, but also, for the people next to them^7^ (protection factors of several respirators can be found in^8^ and information about filter efficiency in^9^). Modeling the potential for wearing face masks (including homemade cloth masks, surgical masks, and FFRs) demonstrated a drastic decrease in peak hospitalizations and deaths, decreasing the SARS-CoV-2 virus's effective transmission rate^10^.

An alarming number of people worldwide question scientific findings and countermeasures against the SARS-CoV-2 virus transmission^11–14^. An early Twitter analysis estimated that around 25% of all tweets regarding the COVID-19 disease contain misinformation^15^. While susceptibility to misinformation seems elevated through social media^16^, COVID-19 related misinformation is shared frequently due to failing to question the content's truthfulness^17^. As such, the potential decline of cognitive performance is discussed. For example, one article concludes that wearing facemasks has physiological and psychological consequences such as—among others—decline in cognitive performance^18^. This is based on a not generalizable finding of declined arterial partial oxygen pressure but unrelated to cognitive performance^19^. However, the manuscript showed several limitations and was, therefore, retracted^20^.

Our study aims to provide clarity and evidence against known myths. We investigated multiple dimensions relevant to cognitive performance. We employ a widely acknowledged questionnaire for mental workload (NASA-TLX^21^) as a subjective assessment. The objective measures are physiological values indicating blood oxygen saturation (SpO2) and heart rate variability (HRV). Regarding the behavioral dimensions, we focus on the number of correctly solved problems within the same time interval, the correctness and response times per trial.

### Related work

Several studies investigated potential physical consequences or health risks caused by face coverings. Several studies showed that wearing a nonmedical face mask does not lead to a decline in oxygen saturation: in older participants during minimal physical activity^22^, no effect on blood and muscle oxygenation in healthy participants^23^, not affecting gas exchange during physical activity for neither healthy nor patients with lung function impairment^24^, and no change in blood oxygen or the heart rate during rest and a flight simulation of healthy pilots wearing N95 FFRs^25^. There were also no differences in heart rate and blood oxygen parameters in health care workers while a one-hour walk wearing N95 masks^26^ and FFR with low filter resistance^27^. However^28^, provides evidence for slightly decreased blood oxygen saturation while wearing N95 respirators for very severe COPD patients. Contrarily, only slight differences in heart rate and pulmonary responses were found in^29^. Perceptions of increased body heat most likely originate from warming of the inhaled air, and the facial skin, skin, and core temperature were not affected by wearing an N95 FFR for more than an hour during physical exercise^30^.

A subjective evaluation of surgeons reported a hampered performance and increased surgical fatigue while wearing FFP2 masks^31^. Also, a decrease in the blood oxygen saturation and an increase in pulse rates before and after wearing masks^32^. Another study compared wearing an FFR(N95) to exercising without one, which did not show significant differences regarding heart rate, respiratory rate, blood pressure, oxygen saturation, or time to exhaustion in a study by Epstein et al., 2020^33^. Solely end-tidal carbon dioxide (EtCO2) levels were increased while wearing an FFR. Other groups compared the physiological effects of exercising with N95 respirators during pregnancy. Both did not find changed heart rate or blood oxygen levels (although diastolic pressure, mean arterial pressure, and subjective exertion)^34,35^.

In an extensive review, several studies investigated the influence of face masks (medical FFR and non-medical face masks) on physiological parameters. They concluded that the effects are negligible and would potentially not impact healthy people even while exercising. However, persons with cardiopulmonary diseases might do experience an effect anyhow^36^.

Deliberate misinformation often uses common knowledge to tell an allegedly fact-based story. Some social media accounts connected heavier breathing while wearing FFR with the false claim to reduce blood oxygen saturation. Indeed, respiration behavior (amongst others, frequency and intensity, see^37^ for a review) changes while wearing an FFR (especially during exercise), and the physical dead volume of the respiratory system causes breathing to be more strenuous^38^. However, there is no evidence that wearing face masks (cloth/surgical masks or FFR) causes the blood oxygen levels to diminish^22–24,26,27,29^. Nevertheless, the literature lacks investigations tailored to quantify the impact of face masks, especially high filtering N95 FFR, on cognitive performance. We contribute to this research to refute misinformation and face worries regarding a connection between cognitive functioning and wearing N95 FFR.

Regarding our variables of interest, findings from Scholey et al., 1999^39^ suggest that in the state of high cognitive demand, the heart rate helps regulate the metabolism, increasing blood oxygen circulation and improving cognitive performance. They showed that oxygen saturation and cognitive performance correlate with each other. Chung et al., 2006^40^ presented similar findings where hyperoxic air administration led to increased blood oxygen saturation and improved accuracy in a verbal cognition task compared to regular air administration. In a different study, the HRV was shown to be sensitive for varying levels of cognitive performance. A higher HRV amplitude is suggested to contribute to a decrease in cognitive performance^41^. Mental stress (e.g., induced by mental arithmetic) decreases the HRV, which is suggested to be a regulation process of the autonomic nervous system^42^.

Additionally, the HRV seems to be a sensitive indicator to discriminate between rest, physical- and mental load. In a study by Tealman et al., 2011 the combination of a physical task (computer mouse work) and a cognitive task (complex arithmetic) showed a significant decrease in HRV features compared to the physical task alone^43^.

The Task Load Index was created to measure demand and the interaction of a subject performing a task^21,44^. It has been frequently used in various like human factors and provides a solid basis for the perceived load.

Behavioral variables are commonly used to measure task difficulty and, thereby, workload. The performance (e.g., measured as a number of solved/correct trials) is expected to decrease when workload reaches a certain threshold^45^. The variation of the difficulty of a task can be indexed in a decrease of correct answers or even in no responses, meaning it was too difficult to solve. At the same time, the duration for producing a response increases if the task is more complex and thereby more mentally demanding than the one before^46^.

### Our contribution

Given the body of evidence, we hypothesize equivalence of blood oxygen saturation while wearing an N95 FFR compared to not wearing one. Further, we hypothesize equivalence of the cognitive demand of the FFR and the no-FFR condition. We expect that the participants perform equally well in both test conditions. In terms of behavioral data, we hypothesize equivalence between the conditions regarding the number of correctly solved tasks, the ratio of correct responses to all tasks presented, the ratio of correct responses to all responses given, the average response time, and the average response time of correct responses.

In terms of physiological data, we assume similarbehavior in both test conditions. The task to be performed has a cognitive focus and is carried out under time pressure. We expect no physical exertion in the relaxed sitting position. Thus, only cognitive demand could influence the physiological parameters as described in the mentioned literature. Providing that cognitive demand is equal in both conditions (with and without an FFR), we hypothesize equivalent results regarding participants' HRV, SpO2, TLX scores, and task performance. This study adheres to CONSORT guidelines.

## Results

**Figure 1:**
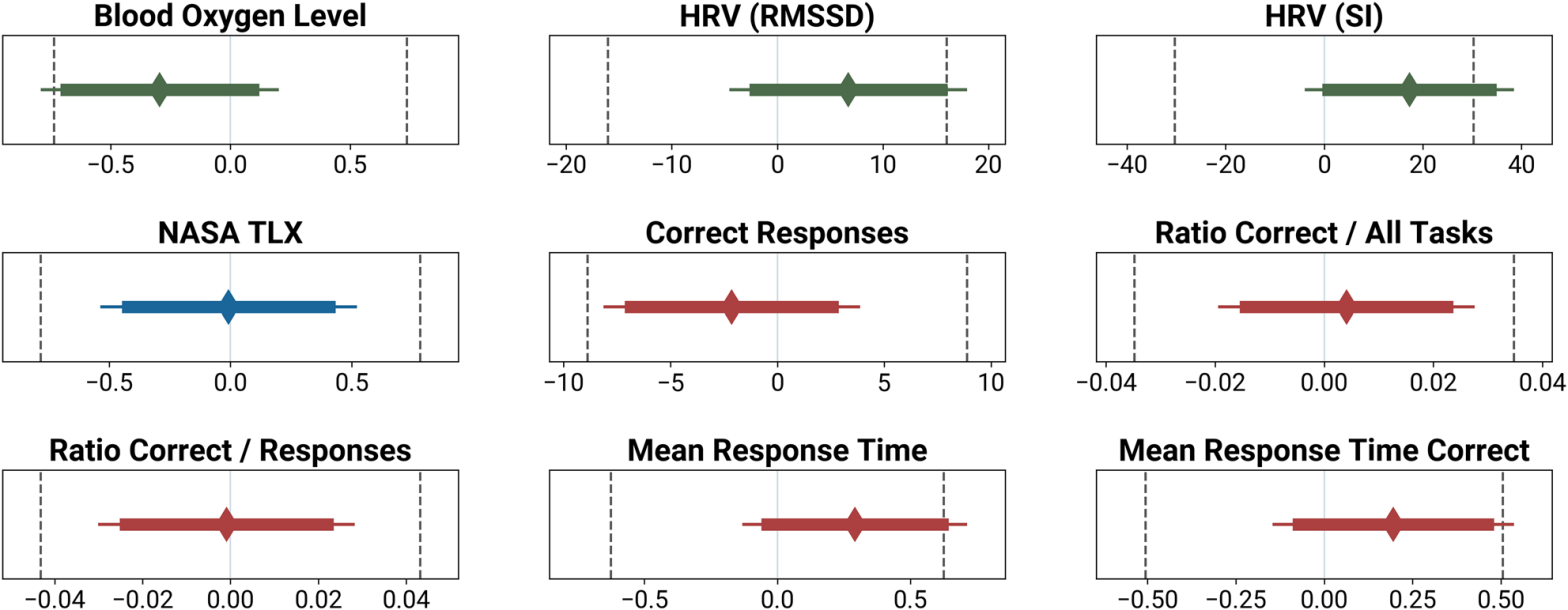
Equivalence boundaries (dotted lines left and right), mean of the mask / no-mask difference (diamond) and the 95% confidence interval (thin line; for the null hypothesis significance test), as well as the 90% confidence interval for the TOST (thick line). The x-axis shows the mean difference in the unit of the metric.

For all following Two One-Sided Test of Equivalence (TOST) procedures, we employed equivalence boundaries of *d*_*z*_ = ±0.45. This smallest effect size of interest (SESOI) translates to the absolute values of the equivalence boundaries reported in the following paragraphs. Figure 1 provides an overview of the TOST confidence intervals and the null hypotheses significance tests, together with the equivalence boundaries.

For both HRV analyses, we had to exclude three datasets due to incomplete recordings. Hence, both are based on data from 41 participants. Given the chosen alpha level of *α* = 0.05 and the pre-defined equivalence bounds of *d*_*z*_ = ±0.45, both HRV TOST results have a statistical power of 1−*β* = 0.78. All other tests are based on all 44 participants, resulting in statistical power of the TOSTs of 1−*β* = 0.82.

### Physiological data

**Figure 2:**
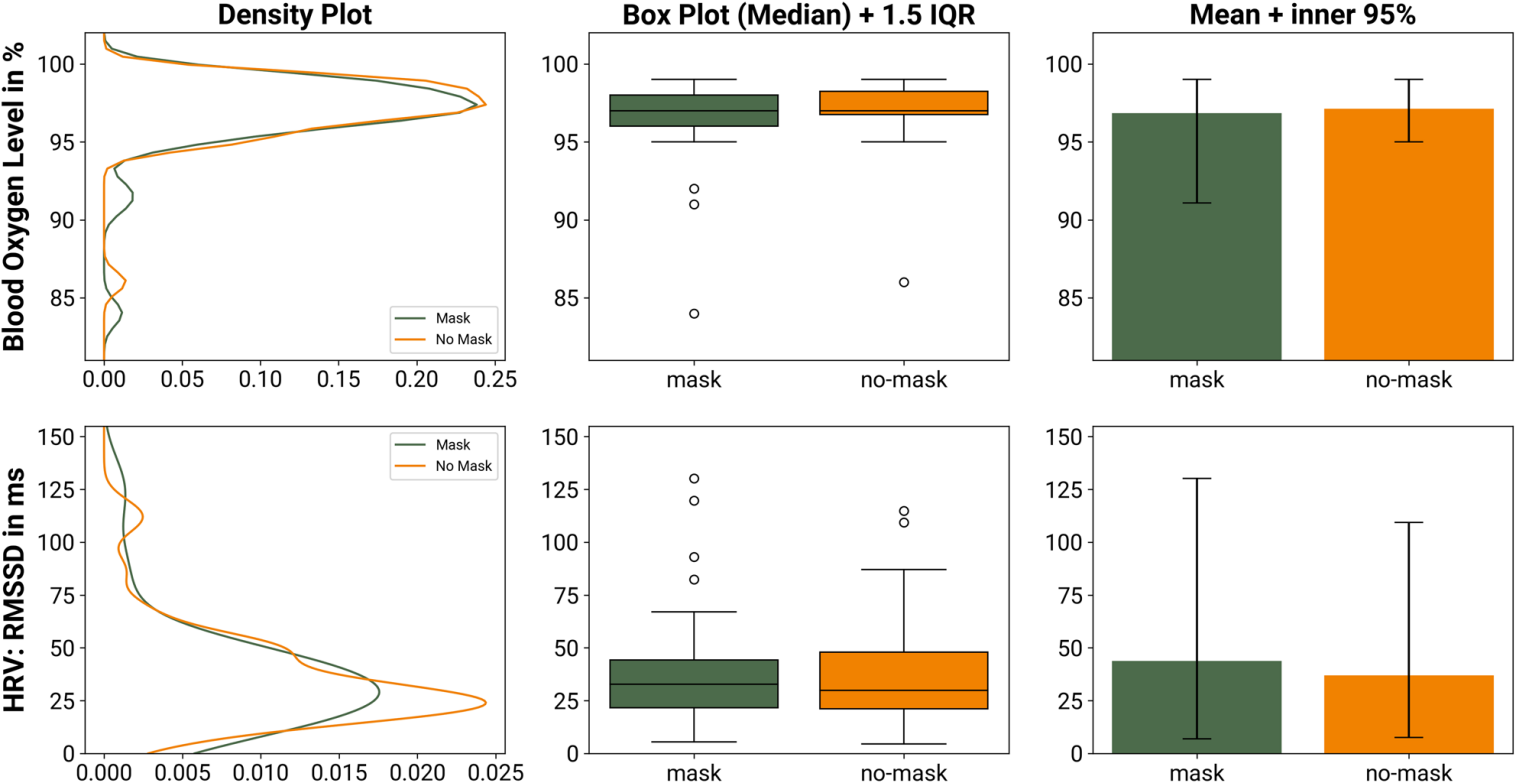
Comparison of the physiological metrics (blood oxygen level and HRV) while wearing an FFR and not wearing an FFR. The density plots to the left describe the similarity of the distributions of the two groups. The box-plots in the center column compare the median and the interquartile range (IQR) and provide an assessment of potential outliers. The bar charts to the right compare the plain mean of the two group; the whiskers depict the inner 95% of the recorded data.

See Fig. 2 for a visualization of the blood oxygen saturation and the HRV measurement (RMSSD) per condition. All result graphs share the same format and visualize different aspects of the group comparison. First, we contrast the distribution of the two groups. For a precise understanding about outliers, centers and spread of the inner 50%, we then align box-plots. In addition to that, we underline the equality of the group means by adding simple bar-plots with 95%-range whiskers.

#### Physiological: blood oxygen levels

The mean difference of blood oxygen level between wearing an FFR (95% CI: 96.04–97.64%) and not doing so (95% CI: 96.48–97.79%) immediately after performing the 15 min of mental calculation is − 0.3% (difference Median: 0%, IQR: 2%). The increase of blood oxygen level without a mask has a negligible effect size of *d*_*z*_ = −0.12. A Shapiro–Wilk test indicated a violation of the assumption of normality (*W* = 0.92, *p* = 0.004). Hence, we employed a robust TOST procedure using Wilcoxon signed-rank test. To compare the measurements of two conditions, we define an equivalence interval. It is derived from our pre-defined effect size of *d*_*z*_ = ±0.45, which translates to ±0.736 in the units of the metric at hand (percent in this case). Hence, the lower equivalence boundary *Δ*_*L*_ = −0.74% and the upper equivalence boundary *Δ*_*U*_ = 0.74%. The TOST procedure reveals that the effect observed is statistically equivalent; the larger of the two p values is less than *α* = 0.05 (*V* = *682*, *p* = 0.014). According to the Neyman-Pearson approach, this means that one can reject the hypothesis that the true effect is greater than *d*_*z*_ = ±0.45 and act as if the effect size falls within these equivalence bounds^47^. According to our pre-registration, we additionally run an exploratory null hypothesis significance test. A pairwise Wilcoxon signed-rank test returned nonsignificant (*V* = 154, *p* = 0.259). Hence the H0 of no difference between groups is not rejected.

#### Physiological: heart rate variability (RMSSD)

The mean difference of RMSSD between wearing an FFR (95% CI: 28.81–58.6 ms) and not wearing one (95% CI: 29.27–44.69 ms) in the last five minutes of each condition is 6.73 ms (difference Median: 1.63 ms, IQR: 11.92 ms). The decrease of the RMSSD without a mask has a negligible effect size of *d*_*z*_ = 0.15. A Shapiro–Wilk test indicated a violation of the assumption of normality (*W* = 0.38, *p* < 0.001), hence we employed a robust TOST procedure using Wilcoxon signed-rank test, with equivalence bounds of *Δ*_*L*_ = –16.06 ms and *Δ*_*U*_ = 16.06 ms. It reveals that the effect observed is statistically equivalent, the larger of the two p values is less than *α* = 0.05 (*V* = *56*, *p* < 0.001). We additionally ran an exploratory null hypothesis significance test. A pairwise Wilcoxon signed-rank test returned nonsignificant (*V* = *519*, *p* = 0.257).

### Subjective data

**Figure 3:**
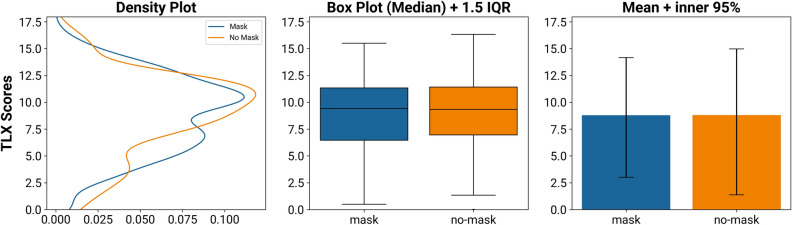
Comparison of the subjective load ratings (NASA TLX) while wearing an FFR and not wearing an FFR. While the distribution reveals minor differences between the groups, these are averaged out when comparing mean and median values.

To investigate the NASA-TLX scores, we first computed the difference between post-task and baseline ratings. The mean difference of these scores is − 0.01 (difference Median: 0.1, IQR: 2.1, see Fig. 3). The decrease of the TLX score without an FFR (95% CI: 7.66–9.95) has a negligible effect size of *d*_*z*_ =  − 0.002 (95% CI of the mask condition: 7.74–9.85). The assumption of a normal distribution was not rejected (*W* = 0.99, *p* = 0.919), so we used a TOST procedure based on Welch’s paired t-test with equivalence bounds *Δ*_*L*_ = −0.78 and *Δ*_*U*_ = 0.78. It reveals that the effect observed is statistically equivalent, the larger of the two p values is less than *α* = 0.05 (*t*(43) = 2.96, *p* = 0.003). An exploratory null hypothesis significance test (pairwise Welch’s t-test) returned nonsignificant (*t*(43) = −0.03, *p* = 0.977).

### Behavioral data

**Figure 4:**
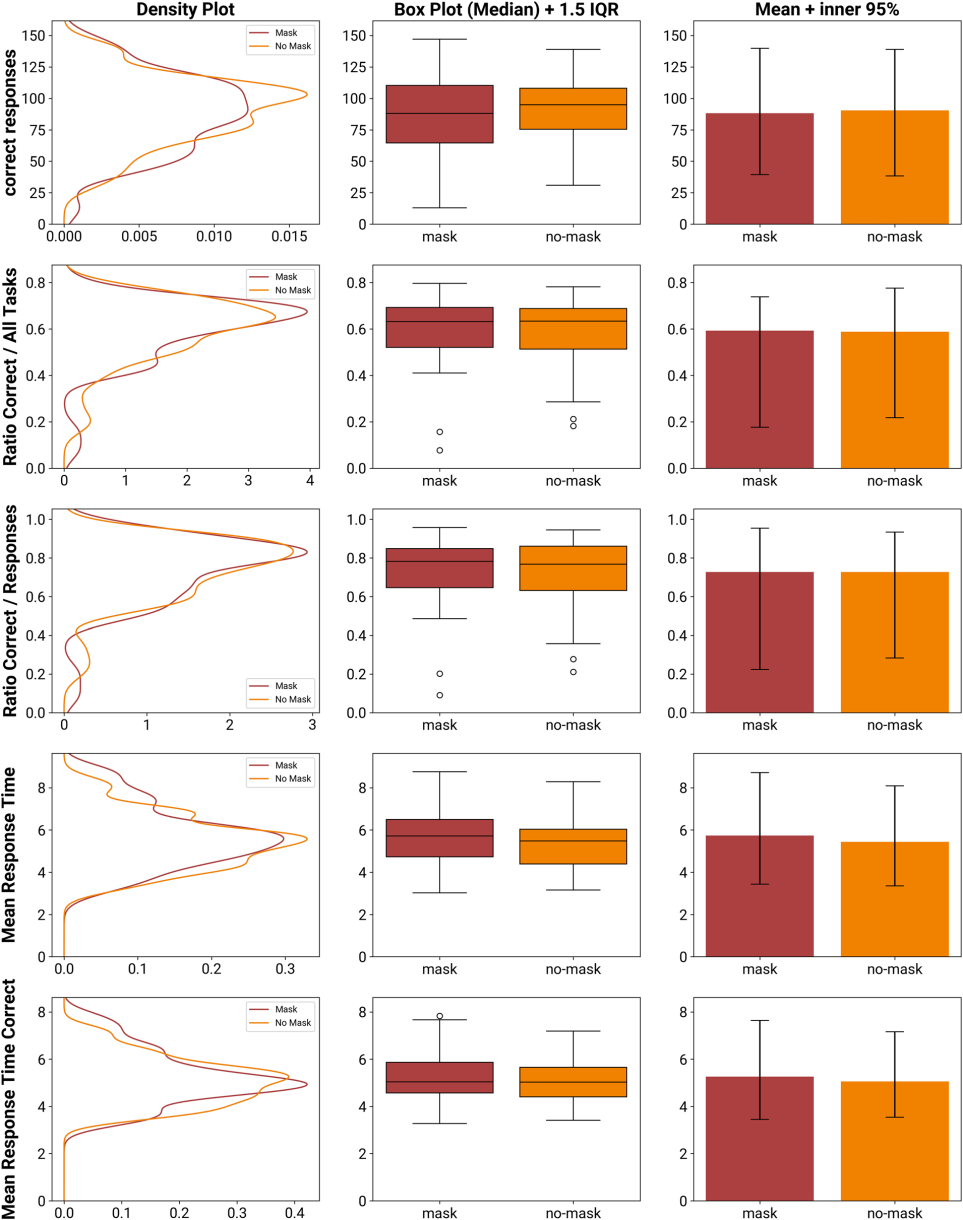
Comparison of the behavioral measures while wearing a mask and not wearing a mask. The dimensions compared are the absolute number of correct responses, the ratios of correct responses against all tasks presented as well as against the number of responses, the mean response time per task, and the mean response time of only the correct responses.

See Fig. 4 for a visualization of the following five behavioral performance data per condition.

#### Behavioral: correct responses

The mean difference between the number of correct responses while wearing an FFR (95% CI: 79.13–97.37) against while not wearing one (95% CI: 82.38–98.39) is −2.14 (difference Median: 3.5, IQR: 24.5). The increase of correct responses in conditions without an FFR has a negligible effect size of *d*_*z*_ = −0.08. The assumption of a normal distribution was rejected (Shapiro–Wilk test, *W* = 0.94, *p* = 0.015), so we used a robust TOST procedure based around the Wilcoxon signed-rank test with equivalence bounds of *Δ*_*L*_ = −8.89 and *Δ*_*U*_ = 8.89. It reveals that the effect observed is statistically equivalent (*V* = 680, *p* = 0.016). An exploratory null hypothesis significance test (pairwise Wilcoxon signed-rank test) returned nonsignificant (*V* = 496, *p* = 0.995).

#### Behavioral: ratio correct responses/all tasks

We investigate the ratio of correct responses against the number of all responses given (correct and incorrect). The mean difference between an FFR and no FFR is nearly zero (difference Median: − 0.01, IQR: 0.09). The effect induced by the FFR (95% CI: 0.55–0.64) is negligible (*d*_*z*_ = 0.03, 95% CI of the no-FFR condition: 0.55–0.63. The assumption of a normal distribution was not rejected (*W* = 0.96, *p* = 0.133), so we used a TOST procedure based on Welch’s paired t-test with equivalence bounds *Δ*_*L*_ = −0.04 and *Δ*_*U*_ = 0.04. It reveals that the effect observed is statistically equivalent, the larger of the two p values is less than *α* = *0.05* (*t*(43) = −2.64, *p* = 0.005). An exploratory null hypothesis significance test (pairwise Welch’s t-test) returned nonsignificant (*t*(43) = 0.35, *p* = 0.728).

#### Behavioral: ratio correct responses/responses given

Next, we investigate the ratio of correct responses against the number of all tasks presented.

The mean difference between FFR and no FFR is nearly zero (difference Median: − 0.01, IQR: 0,1). The effect induced by the FFR (95% CI: 0.67–0.78) is negligible (*d*_*z*_ = −0.01, 95% CI of the no-FFR condition: 0.68–0.78. The assumption of a normal distribution was not rejected (*W* = 0.98, *p* = 0.524), so we used a TOST procedure based around Welch’s paired t-test with equivalence bounds of *Δ*_*L*_ = −0.04 and *Δ*_*U*_ = 0.04. It reveals that the effect observed is statistically equivalent, the larger of the two p values is less than *α* = 0.05 (*t(43)* = 2.92, *p* = 0.003). An exploratory null hypothesis significance test (pairwise Welch’s t-test) returned nonsignificant (*t*(43) = −0.06, *p* = 0.950).

#### Behavioral: mean response time

The mean difference between a mask and no mask of the average response time is 0.29 s (difference Median: − 0.05 s, IQR: 1.46 s). The decrease of the response time in conditions without an FFR (95% CI: 5.06–5.82 s) has a small effect size of *d*_*z*_ = 0.21 (95% CI of the FFR condition: 5.29–6.17 s). The assumption of a normal distribution was rejected (Shapiro–Wilk test, *W* = 0.91, *p* = 0.002), so we used a robust TOST procedure based around the Wilcoxon signed-rank test with equivalence bounds of *Δ*_*L*_ = −0.63 s and *Δ*_*U*_ = 0.63 s. It reveals that the effect observed is statistically equivalent (*V* = 329, *p* = 0.026). An exploratory null hypothesis significance test (pairwise Wilcoxon signed-rank test) returned nonsignificant (*V* = 529, *p* = 0.699).

#### Behavioral: mean response time of correct responses

Lastly, we investigate the average response time of only correct responses. The mean difference between an FFR (95% CI: 4.9–5.6 s) and no FFR (95% CI: 4.76–5.35 s) is 0.2 s (difference Median: − 0.03 s, IQR: 0,93 s). The decrease of the response time in conditions without an FFR has a negligible effect size of *d*_*z*_ = 0.18. The assumption of a normal distribution was rejected (Shapiro–Wilk test, *W* = 0.93, *p* = 0.009), so we used a robust TOST procedure based around Welch’s paired t-test with equivalence bounds of *Δ*_*L*_ = −0.51 s and *Δ*_*U*_ = 0.51 s. It reveals that the effect observed is statistically equivalent (*t*(43) = −1.83, *p* = 0.037). An exploratory null hypothesis significance test (pairwise Welch’s t-test) returned nonsignificant (*t*(43) = 1.15, *p* = 0.255).

## Discussion

The blood oxygen saturation shows a slight decrease of 0.3% after wearing an FFR. This effect is statistically insignificant. Although some discussions against the use of facial masks argue that FFR would impair the body's oxygen supply, this is unstrained by our findings. Instead, we found statistical equivalence and no difference between the test conditions. The HRV metric (RMSSD) showed statistical equivalence when comparing the FFR against the no-mask condition and no significant difference from each other. The HRV seems to decrease slightly (statistically insignificant) in the no-FFR condition on a descriptive level.

When interpreting the HRV metrics as mental load indicators, the RMSSD typically drops if the participant is more strained^48^. On a descriptive level, we find opposing results: the RMSSD indicates slightly more strain, higher intensity load, and focus in the no-FFR condition. This underlines that the changes induced by the FFR cause less variability than the HRV can interpret reasonably.

The subjective NASA-TLX ratings show that the participants perceived a statistically equivalent workload between wearing an FFR and not wearing one. This result may come as a surprise: Because we did not include a blinding protocol, participants were always fully aware of wearing an FFR and not. We did not explicitly tell them about our research question before the experiment was over. However, some participants might have figured out why to wear an FFR sometimes and why not (none of the participants implied so). Nevertheless, because we cannot rule out the possibility of the participants guessing our research question and perhaps even being biased towards governmental pandemic restrictions, it remains possible to have recorded biased results. For this very reason, it seems remarkable that the subjective TLX ratings show no evidence of favoring one of the conditions, not even on a descriptive level. Mainly since the subjective assessment includes an item asking for physical demand that might capture aspects such as wearing comfort (e.g.,^49^ reported "marked discomfort" of the participants wearing FFP2 masks, although the study, in general, is heavily debated, see^50,51^. Other subjective reports also mentioned comfort limitations, e.g., discomfort if the wearer has facial hair^52^ or the problem of subjective difficulty to breathe^53^). Therefore, it seems to be an even greater confirmation that wearing an FFR does not limit the wearer's performance. We deem it unlikely to confound all our different metrics regarding the possible condition awareness, primarily since we investigated a broad spectrum of varying measurement dimensions.

Regarding the behavioral data, the FFR’s influence reached a small effect (*d*_*z*_ = 0.214) for the mean response time; for all other parameters, the effect was negligible. However, this small effect is a statistical artifact that could not be shown to cause a statistical difference. Moreover, the variability induced by the FFR is equivalent to the variability of not wearing one (given our pre-defined equivalence bounds). This means that neither did the participants solve more tasks in 15 min when not wearing an FFR, nor was their correct response ratio any better. Even the average response time was statistically equivalent to the FFR condition. Hence, we deem these findings to refute the claim that facial masks potentially reduce cognitive performance in a meaningful magnitude.

While the participants sat alone in the lab room, not wearing an FFR in one condition, we decided to provide them with N95 FFR ( CE-certified FFP2 in Europe / KN95 in China) for the FFR condition. FFRs generally sit tighter on the face, suffer from less face seal leakage, and its filter medium offers more substantial filter characteristics than surgical masks^54^. Since most homemade masks have even less powerful filtering properties than surgical masks^55^, two interpretations can be drawn for wearers of these more superficial masks: Either the FFR itself primarily attributes the effects found. Then one could suggest that the impact would be diminished even further when wearing surgical or homemade masks. Alternatively, the observed effects are simple non-systematic measurement artifacts. In this case, one would observe effects in the same order of magnitude and similar variations when replicating our work with surgical and homemade masks. In either case, degradation of cognitive performance is not to be expected from wearing FFRs.

The equivalence boundaries we chose are smaller than the effects reported so far. However, this assessment is somewhat rough since no previous work that we are aware of investigated similar relationships and the reported effect sizes of^33,56^ hat to be converted to standardized Cohen's d. To account for conversion errors, we defined our threshold slightly below the definition of a large effect size (which would be *d*_*z*_ = 0.5). We decided to do this because it compromises meaningfulness and a realistic number of test participants. Nevertheless, this definition is potentially our Achilles' heel: our statistical tests' significance relies heavily on the equivalence boundaries. One could argue that these are just wide enough for all our equivalence tests to turn out significantly. While this is de facto the case, it is essential to point out that we pre-defined our equivalence boundaries in the pre-registration before assessing the recorded data and before most of the data has been sampled (as recommended by^47^). However, a replication with smaller equivalence boundaries and a larger sample size would further substantiate our findings.

Other than that, it is worth pointing out that our mental load condition lasted only 15 min. In discussions with mask-skeptic people, we heard the argument that wearing masks for a whole day would impact cognitive functioning. Our comparison based around two 15 min conditions cannot easily be compared to a whole day. However, it is known from the literature that the time in which a change of inhaled air is reflected in blood oxygen readings lies within several seconds up to a minute (e.g.^57^. Hence, if there is no evidence for any impact of the masks after wearing them for 15 min, there is little reason to believe that this drastically changes after several hours.

## Methods

### Task

The main task consisted of solving basic arithmetic equations (addition, subtraction, multiplication, or division) presented visually. Each equation was composed of two numbers (1 to 3 digits) and one operator. All results were positive integers. We decided to implement mental arithmetic because these tasks are suitable for inducing cognitive processing^58^, and the task allows usto vary difficulty levels of the task^59,60^. Additionally, this stimulus is suited to simulate office work^43,61^.

The response time for typing in the correct arithmetic solution was limited, depending on the estimated difficulty of the task. This estimation was done using a prediction model based around Thomas' Q-value, 1963^62^ to estimate a primary arithmetic task's difficulty. This task design allowed us to induce a constant, high mental load for each condition's duration.

### Procedure

We experimented in a small and bright lab room at the Technical University of Berlin during regular office hours. The pandemic situation forced us to limit the time spent with the participants to < 15 min. Hence, the general introduction was done in a separate room, and the time together was spent instructing the conditions. Before and after each experiment, the lab room was heavily ventilated, and all surfaces and devices were disinfected. Due to the strict regulations, the entire floor was hardly occupied, which guaranteed a quiet environment.

The participants were equipped with the chest strap (model: Polar H10; Polar Electro Oy). The ECG data (HR and RR-intervals) was recorded directly via Polar's Bluetooth API to a dedicated smartphone running our recorder app. Additionally, they wore a Comtec Pulse Oximeter, model CMS 50D, which we put on the participant's index finger of the non-dominant hand. The device was active throughout the whole experiment. The FFR we provided was unvented FFP2 NR N95 / KN95 (model number: B13086; Samding Craftwork Co., LTD, Jinniu Daojiao Dongguan Guangdong, China) with a full CE certification (CE 2163, EN 149: 2001 + A1: 2009). Every provided FFR came in a standard size. Since all subjects were adults, we did not use a custom FFR size. However, we instructed the subjects to put on the mask in a well-fitting manner via the adjustable nose clip.

The stimulus presentation was done via smartphone (iPhone XR; Apple Inc.; 6′1-inch screen).

The stimulus presentation was implemented as an iOS app. From top to bottom the app displayed the task, the time left together with a shrinking progress bar, and a key pad to enter the solution and two buttons to delete and to confirm the response.

The measurement of the pulse oximeter is continuous. Therefore, we instructed the participants to note down the current reading of the oximeter right after each test condition.

At the start of the experiment, we conducted a baseline measurement of HRV and subjective data. Therefore, participants filled in a NASA-TLX rating in which they rated their current situation (e.g., waiting in the foyer). Additionally, the participants performed their first blood oxygen measurement as the experimenter showed them beforehand. The baseline recording was also a practice to do all the measurements correctly and took about 5 min in total.

**Figure 5:**
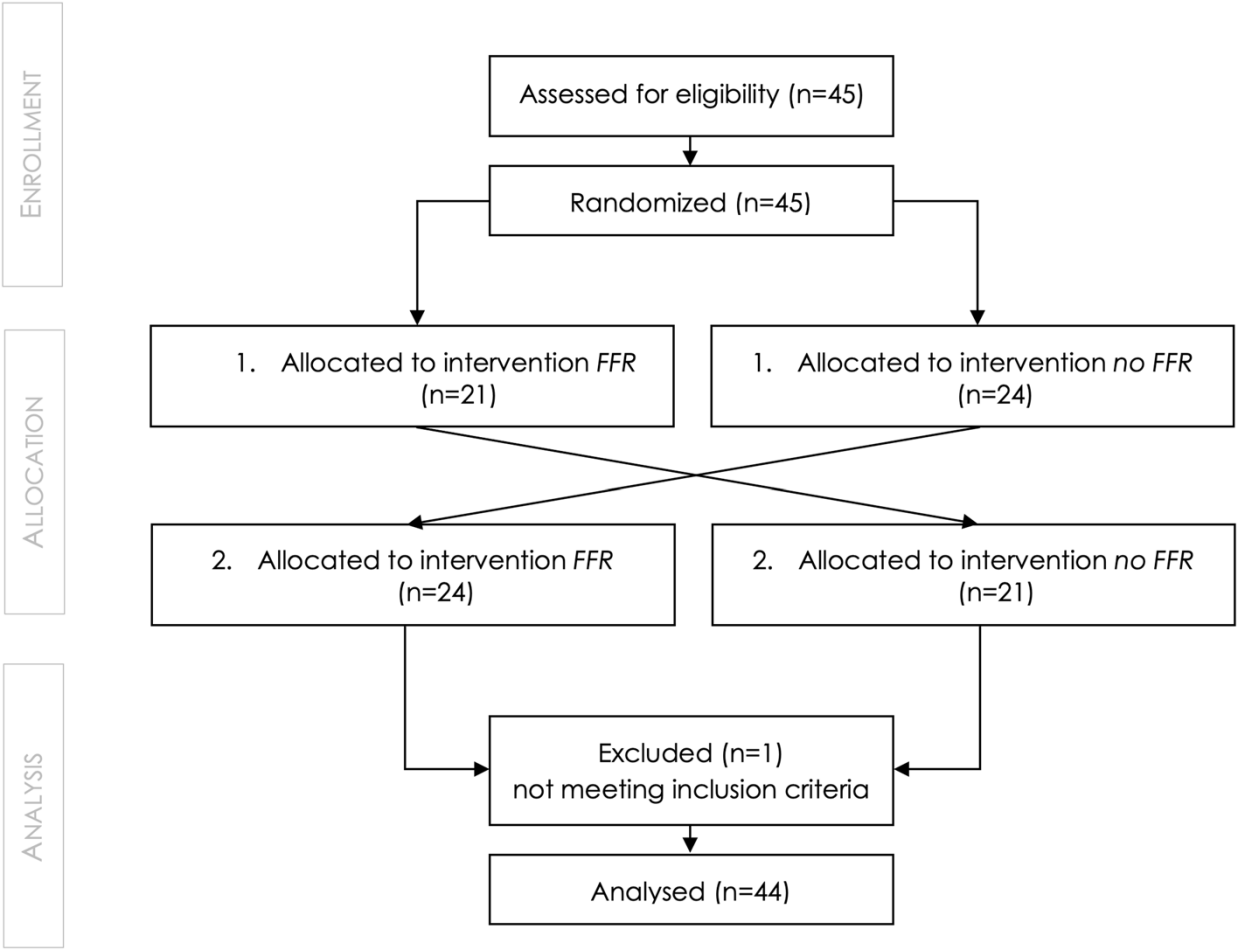
Flow-Chart of the participant flow through the data acquisition.

Each participant was assigned to a random order of the conditions on arrival (see Fig. 5). This randomization was not known to the experimenters before the start of the data collection. The study app announced which condition came next (and logged this to a log file). We used the Swift 5 standard library to generate a random order of the conditions. In one test condition, they performed the arithmetic calculations while wearing a mask, in the other without a mask. Both test conditions had a duration of 15 min. After each condition, the participants had to fill in the NASA-TLX ratings and ran the blood oxygen measurement manually.

After the completion of both conditions, including both post measurements, the experimenter removed the sensors. The participants confirmed the monetary compensation with a receipt.

### Participants

The conduction of the study took place between October and November 2020. An a priori power analysis for matched pairs TOST (alpha = 0.05, power = 0.8, equivalence bound dz =  ± 0.45, cf.^63^) resulted in a minimum required number of 43 participants. We recruited 45 participants to account for possible exclusions due to a lack of correct responses. Twenty-four of the 45 participants identified themselves as female. The mean age was 30.3 years (Median: 29y, IQR: 8y, ranging from 20 to 64y). Twenty-four of the participants hold at least one academic degree; Twenty-two participants were currently enrolled, students. The majority of participants were recruited via the university participant database. It ensures that the offered studies are only visible to people who match predefined criteria, so (usually) no one has to be excluded later on. The only criteria we employ are being aged between 18 and 65, being fluent in German, and having normal or corrected to normal vision. Participants got a monetary compensation of a fixed amount of 12 Euro plus a performance-dependent addition of up to 6 Euro. Besides, some colleagues declared themselves willing to participate. The study protocol was approved by the ethics committee of Technical University Berlin, Faculty IV Electrical Engineering and Computer Science (ethics ID: FT_2020_11). The conductance of the experiment was according to the declaration of Helsinki. The participants obtained informed consent in written form and declared their agreement with the procedure by signature before the recordings began.

The study was a randomized controlled trial. We employed a crossover study design with 45 participants (see Fig. 5). All participants were unaware of the conditions and the differences that we are investigating. However, since they are being told to wear or not wear an FFR before a condition started, they potentially could guess the mask itself is a manipulated factor. One participant had to be excluded from our dataset due to our exclusion criterion defined in the pre-registration. The subject did not reach a minimum performance of min 10% correct trials in the task, which was necessary to be considered in the analysis. So, we considered 44 participants in our general analysis.

### Statistical analysis

Equivalence tests examine whether the presence of large enough effects to be considered meaningful can be rejected^47^. This TOST procedure compares an observed distribution against the boundaries of a predefined equivalence interval. The statistical procedure is then identical to two one-sides t-tests (or equivalent) for determining if the distribution at hand is significantly below the upper equivalence interval boundary, and if it is as well significantly above the lower equivalence boundary. The procedure is thoroughly described by Lakens and colleagues^47^.

In our case, the equivalence test is used to examine whether the difference between wearing an FFR and not wearing one is at least as extreme as a mid-sized effect of *d*_*z*_ = ±0.45.

We defined the SESOI as *d*_*z*_ = ±0.45, based on analyzing reported effect sizes of the related literature (especially^33,56^). However, previous studies had a slightly different focus, so we assumed a slightly smaller effect size than what the colleagues reported. Hence, we decided to choose a fixed effect size just below the “large effect” -guideline *d*_*z*_ = ±0.5. Our definition of the SESOI was part of our pre-registration.

Depending on whether the data is normally distributed, we employ a TOST procedure based on Welch’s t-test (“TOSTER” package v0.3.4 for R), or on the Wilcoxon signed-rank test with continuity correction (“stats” package v3.5.1 of R).

Regarding the HRV measures we binned the time span of each condition into five minutes intervals. For the statistical analysis we computed RMSSD and SI measures for the last interval of each condition only. This way, variations in HRV induced by the onset of each condition should be diminished.

## Conclusion

We hypothesized that wearing an FFR while performing a demanding, cognitive task for 15 min does not statistically differ from completing the same task without an FFR. To do so, we created a testbed allowing us to measure physiological changes in blood oxygen level and heart rate variability, subjective assessment of the mental load, and behavioral performance data. All our findings support all our hypotheses. All metrics recorded with an FFP2 mask are statistically equivalent to not wearing a mask, given our pre-defined equivalence interval of *d*_*z*_ = ±0.45. We interpreted that we can reject the hypothesis of a large effect induced by an FFR (larger than *d*_*z*_ = 0.45). In addition to the statistical equivalence test, we did not find any statistical differences between the two groups. We provided a direct comparison between wearing an FFR and not wearing one. The combination of physiological, subjective, and behavioral data delivers a measurement tool that allows us to detect potential differences objectively and subjectively. Out of that, we are confident that our results support previous research findings and deliver valuable contributions, especially in terms of the current mask debate.

## Data Availability

The datasets generated during and/or analyzed during the current study, as well as the analysis scripts themselves, are available in the Open Science Framework repository: https://osf.io/c2xp5.
